# Depletion of Lipid Efflux Pump ABCG1 Triggers the Intracellular Accumulation of Extracellular Vesicles and Reduces Aggregation and Tumorigenesis of Metastatic Cancer Cells

**DOI:** 10.3389/fonc.2018.00376

**Published:** 2018-10-10

**Authors:** Yuri Namba, Chiharu Sogawa, Yuka Okusha, Hotaka Kawai, Mami Itagaki, Kisho Ono, Jun Murakami, Eriko Aoyama, Kazumi Ohyama, Jun-ichi Asaumi, Masaharu Takigawa, Kuniaki Okamoto, Stuart K. Calderwood, Ken-ichi Kozaki, Takanori Eguchi

**Affiliations:** ^1^Department of Dental Pharmacology, Graduate School of Medicine, Dentistry, and Pharmaceutical Sciences, Okayama University, Okayama, Japan; ^2^Department of Oral and Maxillofacial Radiology, Graduate School of Medicine, Dentistry, and Pharmaceutical Sciences, Okayama University, Okayama, Japan; ^3^Department of Oral Pathology and Medicine, Graduate School of Medicine, Dentistry, and Pharmaceutical Sciences, Okayama University, Okayama, Japan; ^4^Advanced Research Center for Oral and Craniofacial Sciences, Graduate School of Medicine, Dentistry, and Pharmaceutical Sciences, Okayama University, Okayama, Japan; ^5^Department of Oral Diagnosis and Dentomaxillofacial Radiology, Okayama University Hospital, Okayama, Japan; ^6^Division of Molecular and Cellular Biology, Department of Radiation Oncology, Beth Israel Deaconess Medical Center, Harvard Medical School, Boston, MA, United States

**Keywords:** ABCG transporter, ABCG1, tumoroids, extracellular vesicle, metastatic cancer, hypoxia

## Abstract

The ATP-binding cassette transporter G1 (ABCG1) is a cholesterol lipid efflux pump whose role in tumor growth has been largely unknown. Our transcriptomics revealed that ABCG1 was powerfully expressed in rapidly metastatic, aggregative colon cancer cells, in all the ABC transporter family members. Coincidently, genetic amplification of *ABCG1* is found in 10–35% of clinical samples of metastatic cancer cases. Expression of ABCG1 was further elevated in three-dimensional tumoroids (tumor organoids) within stemness-enhancing tumor milieu, whereas depletion of ABCG1 lowered cellular aggregation and tumoroid growth *in vitro* as well as hypoxia-inducible factor 1α in cancer cells around the central necrotic areas in tumors *in vivo*. Notably, depletion of ABCG1 triggered the intracellular accumulation of extracellular vesicles (EVs) and regression of tumoroids. Collectively, these data suggest that ABCG1 plays a crucial role in tumorigenesis in metastatic cancer and that depletion of ABCG1 triggers tumor regression with the accumulation of EVs and their derivatives and cargos, implicating a novel ABCG1-targeting therapeutic strategy by which redundant and toxic substances may be accumulated in tumors leading to their regression.

## Introduction

The mechanism underlying unlimited tumor growth and metastasis are unsolved problems in medicine and biology. We have approached these issues by: (i) study of differently metastatic cancer cells (1, 2), (ii) using a three-dimensional (3D) organoid/tumoroid developed in stemness-enhancing medium compared with popularly-used two-dimensional (2D) culture milieu (2), and (iii) characterization of extracellular vesicles (EVs) secreted by metastatic cancer cells (2–4). We have shown that the rapidly metastatic cell line LuM1 robustly expressed matrix metalloproteinase (MMP)-3 and -9, key proteins in invasion and metastasis whose targeting using siRNA powerfully attenuated subcutaneous tumor growth and metastasis *in vivo* (1). Along with determining such key roles of MMP-3/9, we found a distinct transcriptome of the ATP-binding cassette (ABC) transporter family members in the LuM1 compared with slowly or non-metastatic cell lines. ABC family proteins have been shown to transport numerous kinds of molecules, including inorganic anions, metal ions, peptides, amino acids, sugars, and a large number of hydrophobic compounds and metabolites across the plasma membrane, and across intracellular membranes (5, 6). Among most of the 50 ABC genes contained in the human genome, the ABCG1 gene product plays efflux roles for hydrophobic compounds, lipid and cholesterol (5–8). For instance, in arterial macrophages ABCG1 pumps cholesterol out of the cells leading to reverse cholesterol transport to livers (9). ABCG1 also plays a critical role in mediating cholesterol efflux to high-density lipoproteins (HDL) and preventing cellular lipid accumulation (10, 11). Recent studies showed that ABCG1 was crucial for cancer-initiating/stem cell (CIC/CSC) survival of gliomas (12, 13). It was also shown that conventional chemo/radio-therapy only targets rapidly dividing cells and resistant CIC/CSC pool surviving such therapies undergoes tumor relapse with the expression of drug efflux ABC transporters (14). However, roles for ABCG1 in tumor progression have not been completely clarified yet and require further study. Therefore in the present study, we have aimed to investigate whether ABCG1-mediated extracellular vesicle (EV) lipid efflux altered tumor growth.

Transport and metabolism of lipids, lipoproteins and their cargoes are involved in the functions of EVs. EVs are secreted structures surrounded by lipid bilayer membranes containing a variety of molecular cargoes (15–19). Depending on the mechanisms underlying their release and biogenesis, EVs have been classified as exosomes (30–200 nm), microvesicles (MVs) (100–1,000 nm), apoptotic bodies (1,000–5,000 nm), and matrix vesicles (20–23). EVs, and in particular exosomes usually contain tetraspanins including CD9 (24) while cancer exosomes are often enriched with epithelial cell adhesion molecule (EpCAM), which is highly expressed in CIC/CSC (2, 3, 25, 26). Tumoroids with enhanced CIC properties robustly secrete exosomes carrying increased levels of CD9 and EpCAM (2). Tumors may often originate from the transformation of normal stem cells, and cancer cells may include sub-populations of CIC/CSCs (27–29). We also showed that metastatic oral squamous cell carcinoma cells markedly secrete EVs enriched with stemness marker molecule EpCAM, oncogenic EGFR, and stress resistant proteins HSP90s, whose targeting reduced survival of the metastatic cells (3). Recently, it has become clear that EVs play a key role in cell-to-cell communication and participate in a range of biological events (30, 31). Roles for exosomes in the metastatic process are observed not only in their role of setting/preparing the pre-metastatic and metastatic niche (32) but also in inducing a transformation in mesenchymal stem cells with a key role in the tumor milieu (33, 34). We recently showed that anti-EGFR therapeutic antibody cetuximab is secreted with EVs by oral squamous cell carcinoma cells (4), suggesting that cancer cells could secrete redundant and toxic substances using this process. Many studies have examined paracrine or endocrine roles for EVs, but neither autocrine manner of EVs nor their lipid metabolism have yet been deeply investigated. In the present study, we therefore aimed to investigate the autocrine roles of EVs involving ABCG1-mediated EV lipid metabolism using 3D tumoroid model.

Tumor cell lines have been cloned using their abilities to attach to tissue culture plates and thus the most adhesive cells grow on the plates showing flattened morphologies. However, we found that the LuM1 cells particularly form 3D aggregates even on the two-dimensional (2D) tissue culture plates (see later figures). We have shown that cancer cell aggregation led to formation of hypoxic tumoroids with robust secretion of EpCAM-exosomes and marked upregulation of reprogramming and stemness genes as increased CIC/CSC traits (2). A term of cellular “reprogramming” has been major after the development of induced pluripotent stem (iPS) cells (35–37) and more recently used in cancer studies as well (38–42). Intra-tumoral hypoxia, as well as a hypoxic milieu, can increase CIC/CSC properties through the hypoxia-inducible factor 1α (HIF-1α)-mediated induction of CIC/CSC genes, oncogenes, and ABC transporter genes (2, 43–45). In the central area of tumors when enlarged, elevated HIF-1α reprograms glycolysis and subsequent stemness with increases in reprograming/pluripotency factors such as c-Myc, Oct4, and Lin28 (38).

In the present study, we first aimed to screen and select ABC genes that become powerfully expressed in the metastatic aggregative cells, initially considering all the family members. We then carried out loss-of-function analysis of such a family member- ABCG1, to investigate whether this pump is involved in tumorigenesis with elevated HIF-1α level. We then showed that depletion of the ABCG1 pump triggers the accumulation of autocrine EVs that could trigger regression of malignant tumors.

## Methods

### Cells

A murine colon adenocarcinoma cell line Colon26, its low-metastatic subline NM11, and high-metastatic subline LuM1 were maintained in RPMI1640 supplemented with 10% FBS, penicillin, streptomycin, and amphotericin B in tissue culture plates or dishes (the 2D culture condition) (2, 46). For photomicrography, a Floid cell imaging station (ThermoFisher Scientific, Waltham, MA), BZ-X700 fluorescence microscope (Keyence, Osaka, Japan), and Array Scan High Content Screening System (Thermo, Waltham, MA) were used. For 3D culture milieu, cells were cultured in NanoCulture Plates (NCPs) (MBL Corporation, Nagoya, Japan) unless otherwise specified (2, 47). For stem-cell conditions, cells were cultured in mTeSR1 medium (Stem cell technologies) containing LiCl (1 mM), basic FGF (100 ng/ml), TGF-β (23.5 picomole), GABA (1 mM), insulin (4 μM), transferrin (0.137 μM), β-mercaptoethanol (0.1 mM), cholesterol (1.12 μM), lipids, and BSA in the DMEM/F12 basal medium (48).

### Quantification of hypoxia levels and cellular aggregates

As described by Eguchi T. et al. (2), hypoxia probe Lox-1 (MBL Corporation, Nagoya, Japan) was added at a final concentration of 2 μM the day before the measurement. For analysis of hypoxic cell aggregates in the 2D milieu, 10 k cells were seeded in 96-well 2D culture plates and hypoxia levels were measured 5 days later. For analysis of spheroids and cell aggregates in the 3D milieu, cells were seeded at a concentration of 4,000–10,000 cells/well in 96-well NanoCulture Plates (NCP) (MBL Corporation, Nagoya, Japan) and sizes of cell aggregates were measured 3 or 7 days later by using the hypoxia probe. Relative hypoxia levels were measured using Array Scan High Content Screening System (Thermo, Waltham, MA). The fluorescence intensity of each pixel (μm^2^) and per aggregates of the cells was determined using a filter set for TRITC. Fluorescent areas greater than 300 μm^2^ were counted as cellular aggregates, while those less than 300 μm^2^ were not. A single well-area of a 96-well plate was sectionized to 9 fields, and the area of 1 field is 2,632 μm^2^. For counting cellular aggregates, fluorescent intensity and area (μm^2^ = pixel) of each aggregate in the entire cell population or per field were calculated.

### Gene expression profiling and bioinformatics

Cells were cultured for 3 days and total RNA was extracted using the AGPC method with Trizol (Molecular Research Center, Cincinnati, OH). cDNA was synthesized from 0.1 μg of total RNA using a Low Input Quick Amp Labeling Kit (Agilent Technologies, Santa Clara, CA), then hybridized to probes of a SurePrint G3 Mouse GE 8 × 60 K v.2 Microarray system (Agilent Technologies, Santa Clara, CA). To generate heat maps of relative and absolute expression levels, the raw data were input to MeV 4.0 software (http://www.Tm4.org/mev.html). Members of ABC transporter genes were listed from references (8). Hypoxia-related genes were listed (44). CSC marker genes were listed (49, 50). Raw data were submitted to the Gene Expression Omnibus (GEO) database repository; accession ID: GSE97166; colon26, GSM2553008; LuM1, GSM2553009; NM11, GSM2553010.

### RNAi

A mixture of siRNA with four different sequences was used with UU-3′ overhang for both strands. The sequences of sense strands of siRNA ABCG1 were 5′-GAA GAA GGU GGA CAA CAA, 5′-GAU GUG AAC CCG UUU CUU U, 5′-CGA AUC ACC UCG CAC AUU G, and 5′-AGA CAG ACC UGC UCA AUG G. The sequences of non-targeting dsRNA were 5′-UAG CGA CUA AAC ACA UCA A, 5′-UAA GGC UAU GAA GAG AUA C, 5′-AUG UAU UGG CCU GUA UUA G, and 5′-AUG AAC GUG AAU UGCU CAA-3′.

### Transfection

For RT-qPCR, siRNA (50 or 100 nM) was transfected by using DharmaFECT 1 transfection reagent (Thermo Fisher Scientific, Rockford, IL) according to the manufacturer's instruction. Cells were further cultured for 2 days for RT-qPCR analysis. Otherwise, 40 pmol siRNA was transfected into 500 k cells by using NEPA21 electroporator (NEPA Gene, Ichikawa, Japan). The condition of electroporation was optimized by testing several conditions for each cell line and the condition #6 (150 V, 5 ms) or #3 (125 V, 2.5 ms) were used for LuM1 cells. Cells were cultured for 6 days post-electroporation period and then used for subcutaneous injection, western blot analysis, cell proliferation analysis, and cell aggregation analysis.

### RT-qPCR

Relative mRNA levels were quantified as described (51). For quantification of gene expression levels with accuracy, *Hprt1, Hplp0, Hplp4, H3f3a, B2m, Gapdh*, and *Actb* were examined as candidates of internal controls and *Hprt1* and *Hplp0* were used as an appropriate internal control. The following primer sequences were used: murine *Abcg1* Fw, 5′-TTG ACA CCA TCC CAG CCT AC-3′; murine *Abcg1* Rv, 5′-AGC CGT AGA TGG ACA GGA TG-3′; murine *Hprt1* Fw, 5′-TGC TCG AGA TGT CAT GAA GGA G-3′; murine *Hprt1* Rv, 5′-AAT CCA GCA GGT CAG CAA AG-3′; murine *Abcg2* Fw, 5′-TCG CAG AAG GAG ATG TGT TG-3′; murine *Abcg2* Rv, 5′-TTG AAA TGG GCA GGT TGA GG-3′; murine *Hif1a* Fw, 5′-TCA TCA GTT GCC ACT TCC CC-3′; murine *Hif1a* Rv, 5′-ATG TAA ACC ATG TCG CCG TC-3′.

### Western blot analysis

As described (52, 53), protein samples were loaded onto 10% polyacrylamide gel, transferred to a PVDF membrane by using a semi-dry method. Blocking and antibody reactions were done in blocking buffer containing 3% skim milk (Wako, Osaka, Japan) in Tris-buffered saline containing 0.05% Tween 20 (TBS-T). An anti-ABCG1 antibody (N-term) (1:1,000, AP6529A, Abgent, Jiangsu, China) and HRP-conjugated anti-GAPDH antibody (1:5,000, Wako, Osaka, Japan) were used.

### Cell proliferation and viability assay

As described by Eguchi T. et al. (2), cells were seeded at a concentration of 1 × 10^4^ cells/well in a 96-well culture plate (2D) or 96-well NCP (3D) and culture for 7 days. The cells were separated by using Trypsin/EDTA solution and counted by using Countess automated cell counter (Invitrogen, Carlsbad, CA). To examine LuM1 proliferation altered by EVs, recipient LuM1 cells were transfected with ABCG1-targeting or control siRNA and seeded at a concentration of 1 × 10^4^ cells per well in a 96-well plate. The cells were cultured for 24 h in serum-containing medium to which LuM1-derived EVs (LuM1-EVs) were added. NucBlue (ThermoFisher Scientific) was added to the medium and number of cells at 3 hours post-EV addition period was counted using Array Scan High Content Screening System (Thermo, Waltham, MA).

For cell viabilities in tumoroid culture, cells were transfected with 15 pmol/ml of siRNA in a 6-well plate. The transfected cells were detached with trypsin and reseeded in 96-well 2D-culture plates or NCPs at a concentration of 10 thousand cells per well within DMEM contained with 10% serum or mTeSR1 stem cell medium. Cells were detached using trypsin and counted using a Countess cell counter at day 2, 4, and 6 post-seeding periods (*n* = 4).

### Preparation and analysis of EVs

As described by Ono K et al, EVs were prepared using polymer-based precipitation method (3). As described (2, 3), EVs were visualized with 20,000 times magnification with an H-7650 transmission electron microscope (Hitachi, Tokyo, Japan) at Central Research Laboratory, Okayama University Medical School. For particle diameter distribution analysis, a part of EV fraction was diluted within PBS (–) to volume up to 40 μl and then analyzed using Zetasizer Nano ZSP (Malvern Panalytical, Malvern, UK) in a range of 0.3–10,000 nm-diameters (3).

### EV lipid transport analysis

For fluorescence labeling of EVs, 20 μg of EV was incubated with 10 μM BODIPY TR Ceramide (ThermoFisher Scientific) for 20 min at 37°C. Excessive BODIPY TR Ceramide was removed using Exosome Spin Columns (MW 3000) (ThermoFisher Scientific). Cells were grown for 7 days on 2D culture plates after transfection. As recipients, the cells were seeded at a concentration of 1 × 10^4^ cells/well in a 96-well plate and cultured for 24 h in an above-mentioned serum-containing medium. EVs labeled with red fluorescent sphingolipid described above were added to culture media of the recipient cells at the concentration of 1.0 μg/well for 3 or 24 h. Hoechst33342 dye (NucBlue^®;^, ThermoFisher Scientific) was added 1 h before the following analysis. The number of cells and fluorescence were measured by using Array Scan High Content Screening System (ThermoFisher Scientific) as described (2). Filters for TRITC and Hoechst 33342 were used. The integrated fluorescence intensity of each cell was calculated and the intensity of 500 or more was defined as an EV positive cell while those less than 500 were not. For analysis of EV-lipid accumulation in 3D tumoroids, we established and used fluorescent LuM1 cells that stably expressed ZsGreen green fluorescent protein driven by the murine *Mmp9* promoter (−600 to TSS). The cells were transfected with siRNA and reseeded into 96-well NCPs at a concentration of 5 thousand cells per well in mTeSR1 medium. The BODIPY TR ceramide-labeled LuM1-derived EVs were added to the conditioned medium at a concentration of 1 μg per well at day 4 post-seeding period and the accumulation of the EV-lipids were monitored every 30 min for 24 h in the Array Scan system with channel 485/549/bright field).

### Animal experiments

This study was carried out in strict accordance with the recommendations in the Guide for the Care and Use of Laboratory Animals of the Japanese Pharmacological Society. The protocol was approved by the Committee on the Ethics of Animal Experiments of the Okayama University (Permit Number: OKU-2015659). All surgery was performed under sodium pentobarbital anesthesia, and all efforts were made to minimize suffering. For knockdown studies, LuM1 cells were transfected with ABCG1-targeting or control siRNA and cultured for 6 days on 2D plates. Cells were pre-cultured on 2D plates and detached with Trypsin/EDTA. The knockdown was confirmed by Western blot analysis. Cells (5 × 10^5^) were subcutaneously transplanted into a side abdominal wall or on a back of each BALB/c mouse (Japan SLC, Shizuoka, Japan) at 6–7 weeks old. Primary tumors were resected 22 days later, then fixed with 4% paraformaldehyde.

### Immunohistochemistry

As described (2). Antibodies against ABCG1 (AP6529A, Abgent, Jiangsu, China, 1:100), Ki67 (Dako M7248, Clone MIB-5) and HIF-1α (CST D1S7W, 1:800) were used. To quantify positivity rates of ABCG1 and HIF-1α, positivity of 1,000 cells in random five fields around necrotic areas in tumors were measured under high-power field.

### Correlation analysis of gene expression with cancer prognosis

The meta-analysis of correlation between gene expression levels and prognosis of patients were carried out using PrognoScan (54) and survival analyses with Kaplan-Meier plots were obtained.

### Meta-analysis of genetic alteration

To analyze alteration in APC in rectal, colon, colorectal, and mucinous adenocarcinoma, cBioPortal was used. Genetic alteration in ABC-G group genes were analyzed in whole data (48,596 samples in 169 studies) available in cBioPortal version 1/4/2018, the breast cancer patient xenograft study (British Columbia, Nature 2014; 117 samples), the metastatic breast cancer project (Provisional, October 2017; 103 samples), and NEPC (Trento/Cornell/Broad 2016; 114 samples) (55).

### Statistics

Statistical significance was calculated using Microsoft Excel or GraphPad Prism. The comparisons of 2 were done with an unpaired Student's *t*-test unless otherwise specified. The comparisons of 3 or more groups were done with One-way ANOVA with using pairwise comparison by Tukey's multiple comparisons test. Data were expressed as the mean ± S.D. unless otherwise specified. Box-and-whisker plots were expressed with a median (center line), upper and lower quartiles, upper and lower extremes, and a mean as × . Minimum *P*-values, corrected *P*-values, and COX *P*-values were calculated in PrognoScan.

## Results

### ABCG1 and ABCG2 gene expression was elevated in aggregative metastatic cells

**Figure 1:**
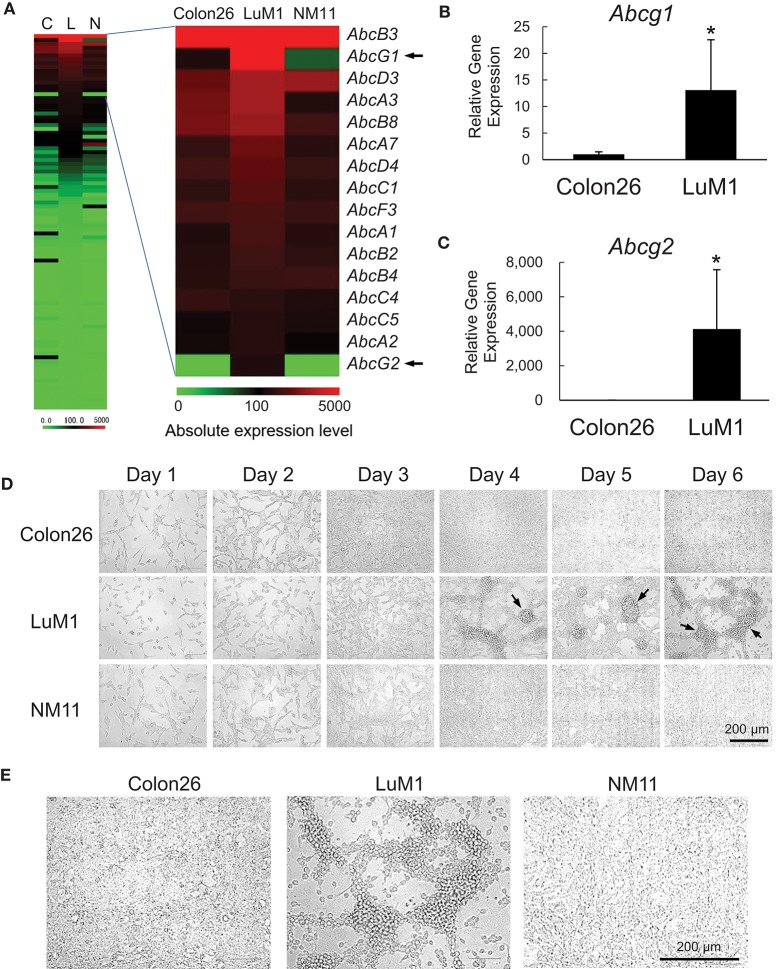
Transcriptome and morphologies of colon cancer cells with different metastatic potentials. **(A)** Heat map analysis of absolute expression levels of ABC genes. Left, all ABC genes. Right, top 16 ABC genes expressed in LuM1 cells. C, Colon26; L, LuM1, N, NM11. **(B,C)** Relative mRNA levels of *Abcg1*** (B)** and *Abcg2*** (C)** analyzed using RT-qPCR. *n* = 4, biological replicates. *p < 0.05 (Mann-Whitney test). The mRNA levels were normalized with the levels of an internal control *Hprt1*. **(D,E)** Representative photomicrographs of LuM1, NM11, and Colon26 cells at day 1 to day 6 **(D)** and day 4 **(E)** post-seeding period. Cells were cultured in a serum-containing medium on 2D culture plates. Scale bar, 200 μm. Arrows indicate cellular aggregates. Enlarged images of cells at day 4 was shown in **(E)**.

We examined the gene expression levels of the ABC genes among the aggregative metastatic cells compared to non-aggregative less-metastatic cells by analyzing their transcriptome. The absolute and relative expression levels of *Abcg1* in LuM1 cells were significantly higher than the two slowly metastatic cell lines, as well as higher than those of a number of the other members of the ABC transporters (Figure 1A). The relative expression level of *Abcg2* was 204-fold higher in LuM1 cells than that in Colon26 cells, although the absolute expression level of *Abcg2* was not at a profound level (Figure 1A). The mRNA levels of ABCG1, as well as ABCG2, were however elevated in the aggregated LuM1 cells compared to non-aggregative Colon26, confirmed by RT-qPCR analysis (Figure 1B,C).

The different metastatic traits of LuM1, NM11, and their parental Colon26 had been shown previously, although their morphological traits have not yet been studied. We therefore next investigated the morphologies of these cell populations *in vitro*. Cells derived from these three lines formed spindle shapes when they were growing sparsely at days 1–3. Of note, however, only LuM1 cells formed 3D-aggregates when they became confluent at days 4–6 (Figure 1D, arrows 1E). These data suggested that the metastatic nodule-forming LuM1 cells had an advanced plasticity with regard to aggregative properties in the 2D culture milieu.

We next confirmed that the differential metastatic traits of these cell lines were retained after being maintained *in vivo*. We injected LuM1 and Colon26 cells subcutaneously into immunologically normal syngeneic mice. The LuM1 injected group showed higher tumor incidence (75 vs. 25%) and the higher number of metastatic nodules (17.8 ± 9.9 vs. 1.0 ± 1.0) compared with the Colon26 injected group (Figure S1). These findings indicated therefore that expression of the *Abcg1* and *Abcg2* was elevated in colon cancer cells with rapidly metastatic and aggregative traits.

### Increased production of ABCG1 and EV, but low level of Ki67 in tumoroids *in vitro*

**Figure 2:**
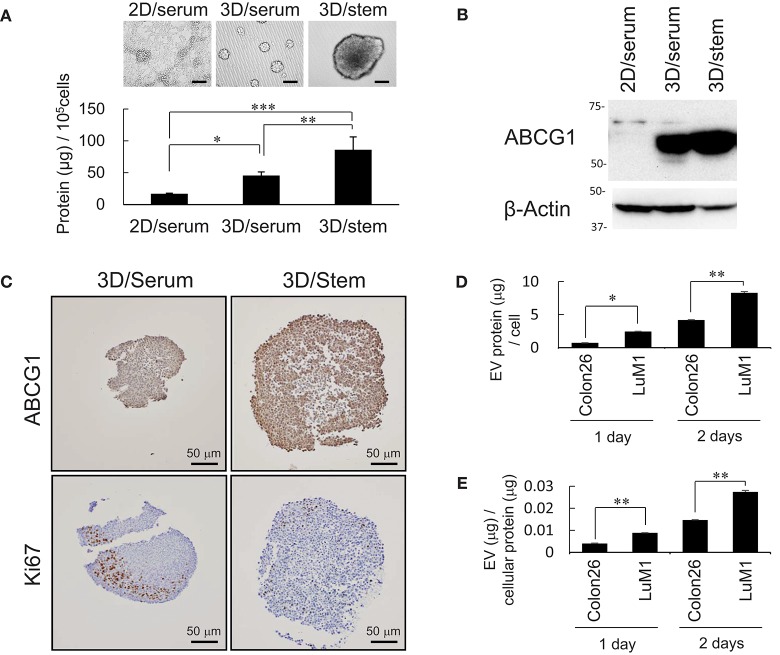
Abundant production of ABCG1 and EV, but low level of Ki67 in tumoroids. **(A)** Representative morphologies (top) and protein concentration (bottom) in tumoroids of LuM1 cultured in 2D/serum, 3D/serum, and 3D/stem conditions. *n* = 3, *P < 0.05, **P < 0.01, ***P < 0.005. Similar data were obtained from at least two independent experiments. **(B)** Western blot showing ABCG1 in tumoroids developed in 3D/stem, 3D/serum, and 2D/serum conditions. β-actin, loading control. Proteins per 10^∧^5 cells were loaded. **(C)** Immunohistochemistry showing ABCG1 and Ki67 in tumoroids. Cell nuclei were counterstained with hematoxylin. Scale bars, 50 μm. **(D,E)** Concentration of EVs in conditioned medium of aggregated LuM1 and Colon26 cells. EVs were prepared from serum-starved conditioned medium for 1 or 2 days. EV protein concentrations per 10^6^ cells **(D)** and per cellular protein concentration **(E)** were measured. *n* = 4, *P < 0.05, **P < 0.01.

It was shown that some CIC with CSC-like properties grow very slowly (2). We have shown that tumoroids of metastatic prostate cancer cells with enhanced stemness produce more proteins and EVs compared with serum-stimulated differentiating cells (2). These findings prompted us to examine whether production of ABCG1, a marker of proliferating cells Ki67, and EV proteins increased in stemness-enhanced tumoroids of the colon cancer cells compared with serum-stimulated differentiating aggregates. The tumoroids formed in the 3D/stem condition produced more proteins than serum-stimulated cells (Figure 2A). Indeed, ABCG1 levels per cells were higher in the tumoroids with enhanced stemness than those in 2D-cultured serum-stimulated differentiating cells (Figure 2B).

To examine expression and localization of ABCG1 and Ki67, immunohistochemistry was performed on stemness-enhanced or serum-stimulated tumoroids. The levels of ABCG1 were higher in stemness-enhanced tumoroids (3D/stem) than those in serum-stimulated tumoroids (Figure 2C, top). In contrast, Ki67 levels were higher in serum-stimulated tumoroids than that in stemness-enhanced ones (Figure 2C, bottom), as expected. ABCG1 was found in whole tumoroids and particularly strong positivity of ABCG1 was found at the marginal area of these structures (Figure 2C, top), suggesting a transporter role for ABCG1 in tumoroids and potentially in tumors. Ki67 was found in the peripheral area of the tumoroids but in neither central area nor the rind area. Thus, it was suggested that ABCG1 could play a transporter role between outside and inside of the tumoroids.

It is known that EVs could transport substances between cells. We therefore next compared EV protein secretion between aggregated LuM1 and flat-cultured Colon26 cells. The aggregated LuM1 cells secreted more EVs (approximately 8 μg EV proteins / a cell; EV vs cell protein ratio = 3: 100) compared with Colon26 cells (Figures 2D,E, Figure S2). However, ABCG1 was not found in the LuM1-derived EVs (data not shown), indicating that ABCG1 plays a role in cells in tumors but is not secreted with EVs. These findings suggested that while production of ABCG1 and EV increased, the number of Ki67 positive, proliferating cells was lowered in tumoroids.

### Targeting of ABCG1 reduced tumor growth *in vitro* and *in vivo*

**Figure 3:**
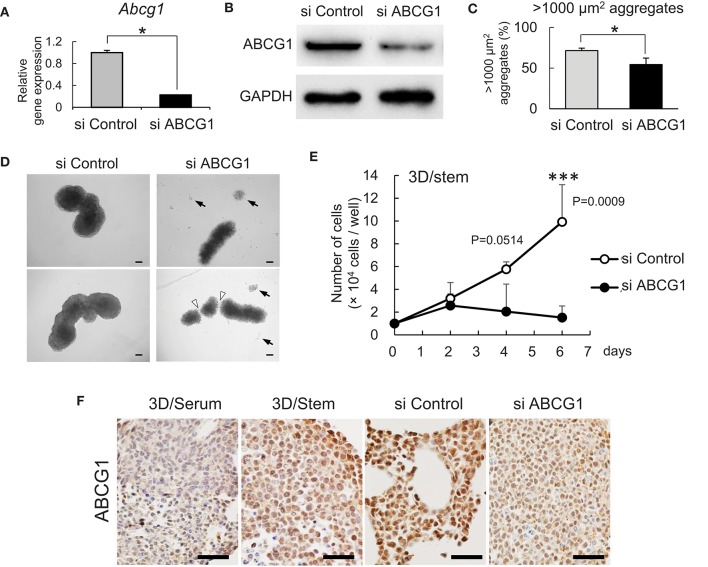
Depletion of ABCG1 attenuates growth of tumors. **(A)** Relative mRNA levels of ABCG1 between the ABCG1-siRNA and the control-siRNA transfected LuM1 cells. The mRNA levels were normalized with the levels of an internal control *Hprt1*. *P = 0.0183, *n* = 3. Similar data were obtained from three independent experiments. **(B)** Western blot showing ABCG1 knockdown in LuM1. The siRNAs were transfected via electroporation. **(C)** The rate of LuM1 aggregates larger than 1,000 μm^2^ in a 96-well plate. *P = 0.0177, *n* = 3. Similar data were obtained from three independent experiments. **(D)** Representative morphologies of tumoroids with or without ABCG1 depletion. Arrows, dead cells dissociated from tumoroids. Arrowheads, gaps between aggregates. Tumoroids in day 5 post-seeding periods were shown. The tumoroids were grown in ultra-low attachment plates. **(E)** Tumoroid cell growth with or without ABCG1 depletion. Tumoroids were grown in the 3D/stem condition. ***P = 0.0009 (day 6). *n* = 4 (biological replicates). Similar data were obtained from two independent experiments. **(F)** Immunohistochemistry showing ABCG1 in tumoroids cultured within 3D/serum or 3D/stem conditions or formed by ABCG1-depleted or control LuM1. Scale bars, 50 μm. ABCG1 positivity was shown on the bottom. Left two photomicrographs were magnified ones of Figure 2C.

To investigate a role for ABCG1 in tumoroids with enhanced stemness, we next optimized efficient knockdown of ABCG1 prior to functional studies. One has experienced that mRNA levels of housekeeping genes such as *Gapdh* and *Actb* were altered according to cellular contexts. Therefore, we asked an appropriate internal control for RT-qPCR and found that *Hprt1* was the one (Figure S3). By using *Hprt1* as an internal control, we confirmed that the expression of ABCG1 at the mRNA level was significantly decreased after transfection of an siRNA pool that targeted *Abcg1* mRNA (Figure 3A). We next established efficient transfection conditions and for depletion of ABCG1 at the protein level. We examined ten conditions of electroporation-mediated transfection and found that particular combinations of voltage and pulse length were most efficient (Figure S4). Using the optimized protocol, the level of ABCG1 was significantly decreased after transfection of the Abcg1-siRNA pool at day 5 after the transfection (Figure 3B, Figure S5). To investigate a role of ABCG1 in the spheroid formation by the metastatic cells, we next examined whether depletion of the transporter could alter LuM1 aggregation on the 3D culture milieu. Indeed, depletion of ABCG1 significantly lowered the rate of formation of cell aggregates larger than 1,000 μm^2^ (Figure 3C, Figure S6).

We next examined whether the morphology and viability of tumoroids were altered by depletion of ABCG1. The control tumoroids maintained viable morphologies while disassembled dead cells appeared around the ABCG1-depleted tumoroids (Figure 3D). Indeed, ABCG1 depletion significantly reduced the viability of the stemness-enhanced tumoroids (Figure 3E).

We next investigated whether ABCG1 level was reduced in the ABCG1 knockdown tumoroids. Tumoroids grown in 3D/stem milieu showed ABCG1 positivity more extensively than the organoids with serum-stimulation. The ABCG1 level in ABCG1-siRNA transfected tumoroids was lower than control siRNA-transfected ones (Figure 3F).

We next examined whether depletion of ABCG1 could alter expression of ABCG2 and the growth of the metastatic cell line LuM1. We confirmed the expression levels of *Abcg1* mRNA were reduced with either 50 nM or 100 nM siRNA-ABCG1 (Figure S6). *Abcg2* mRNA level was reduced with 50 nM siRNA-ABCG1, but not significantly reduced with 100 nM siRNA-ABCG1 (Figure S6). ABCG2 expression might be under the control of ABCG1. However, a complex mechanism was indicated, which we describe in the discussion.

These results indicated that targeting of ABCG1 led to attenuation of tumor growth of the aggregative, metastatic cancer cells.

### Targeting of ABCG1 reduced HiF-1α level in subcutaneous tumors

**Figure 4:**
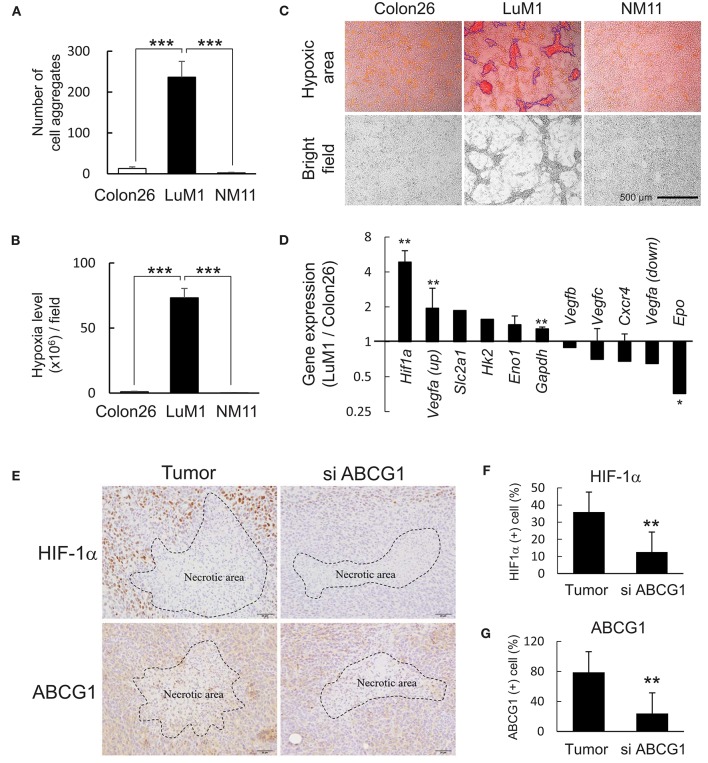
Depletion of ABCG1 declined HIF-1α level in tumors. **(A,B)** Differential abilities to form hypoxic aggregates **(A)** and hypoxia levels **(B)** of LuM1, Colon26, and NM11. ***P < 0.0001 (vs. LuM1), *n* = 4. Similar data were obtained from 10 independent experiments. The number of aggregates per a well in a 96-well plate was shown in **(A)**. **(C)** Representative images of hypoxic aggregates. Top, merged images of hypoxia probe signal (red and orange areas) and bright fields. Fluorescent hypoxic areas greater than 300 μm^2^ were enclosed with blue lines and counted as hypoxic cell aggregates. Bottom, bright fields. Scale bar, 500 μm. **(D)** Expression profile of hypoxia-related genes (LuM1 vs. Colon26). mRNA levels of *Hif1a* and its target genes in LuM1 cells were divided by those in Colon26 cells. **P < 0.01, *P < 0.05. *Hif1a, n* = 3; *Eno1, n* = 3; *Gapdh, n* = 7; *Vegfc, n* = 3; *Cxcr4, n* = 2; *Vegfa* up, *n* = 2; *Vegfa* down, *n* = 2; *Epo, n* = 2 (number of probes used in microarray). **(E)** Representative immunohistochemistry showing HIF-1α and ABCG1 in tumor central area at day 22 post-injection period. Necrotic areas were enclosed with dotted lines. Scale bars, 50 μm. **(F,G)** positivity rates of HIF-1α **(F)** and ABCG1 **(G)** around the necrotic areas in ABCG1-depleted or control tumors. **P < 0.01, *n* = 5.

We next examined HIF-1α levels *in vitro* and *in vivo* and a role of ABCG1 in tumor HIF-1α level. We found that more than 240 aggregates were formed by LuM1 in a 6-well plate while less than 10 aggregates were formed by Colon26 and NM11 cells (Figure 4A). The hypoxia level of LuM1 aggregates was 75-fold higher than that of Colon26 and NM11 (Figure 4B). Indeed, the cell aggregates formed by the LuM1 cells were visualized with the red fluorescence of the hypoxia probe but not in the Colon26 or NM11 cells, indicating that the aggregates formed by LuM1 cells were markedly hypoxic (Figure 4C). As HIF-1α would be predicted to be activated under these hypoxic conditions, we next examined the gene expression profile of *Hif1a* and its target genes in the hypoxic aggregates of LuM1 and the more normoxic Colon26 cells. Gene expression level of *Hif1a* was significantly higher in LuM1 than that in Colon26 cells (Figure 4D). Upregulation of *Vegfa* mRNA (with 2 types of probes) was found in LuM1 cells whereas downregulation of *Vegfa* mRNA (with 2 other types of probes) was also found in LuM1 cells, suggesting that alternative splicing of *Vegfa* might be altered (Figure 4E). Although hypoxia-inducible erythropoietin (*EPO*) gene expression was shown in human neuroblastoma cells (56), downregulation of *Epo* mRNA was found in LuM1 aggregates as compared with Colon26 cells, We next examined whether ABCG1 depletion altered HIF-1α levels in tumors. Strong positivity of HIF-1α and ABCG1 were found close to the necrotic area in tumors (Figure 4E, left) whereas reduced levels of these proteins were found around the necrotic area in the ABCG1-depleted tumors (Figure 4E). We quantified positivity rates of HIF-1α and ABCG1 around the necrotic areas in tumors. The positivity rate of HIF-1α as well as ABCG1 around necrotic areas were significantly reduced in the ABCG1-depleted tumor compared with the control tumor (Figures 4F,G). These findings indicated that targeting of ABCG1 reduced the HIF-1α level around the central necrotic area in tumors, suggesting potential anti-angiogenic effects.

### Depletion of ABCG1 triggered the accumulation of EVs and reduced survival of tumor cells

**Figure 5:**
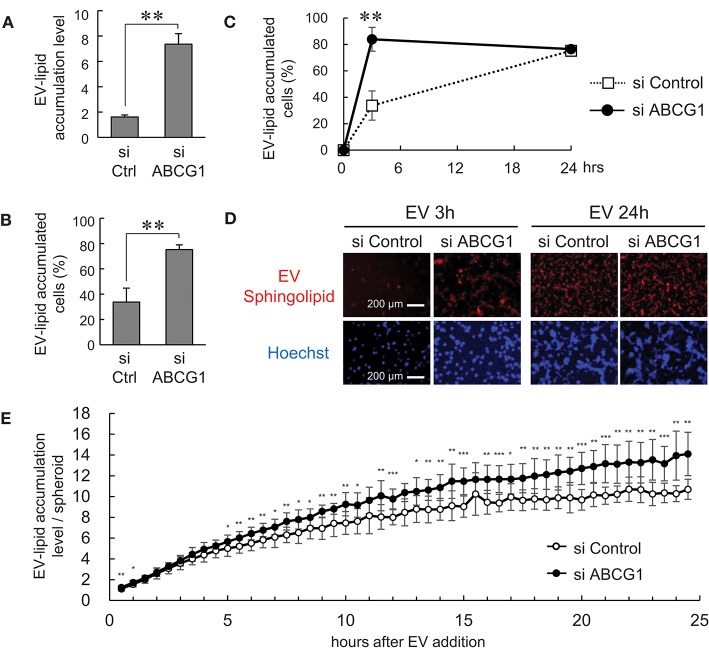
Depletion of ABCG1 triggered accumulation of EVs and declined survival of tumor cells. **(A–E)** Depletion of ABCG1 increased accumulation of EV-derived lipid in LuM1 cells in 2D-culture condition **(A–D)** and 3D tumoroids **(E)**. LuM1-derived EVs were labeled with fluorescent sphingolipid and added to conditioned media of ABCG1-depleted or control LuM1 cells. **(A)** Accumulation levels of EV-lipid. Accumulation levels per cell at 3 h post-EV addition period were shown. **P = 0.0004, *n* = 4 (biological replicates). **(B)** The rate of EV-lipid-accumulated cells. The rate of EV-lipid accumulated cells at 3 h post-EV addition period was shown. **P = 0.0003, *n* = 4 (biological replicates). **(C)** Kinetics of EV accumulation in the 2D-cultured LuM1. **P = 0.0004, *n* = 4 (biological replicates). **(D)** Representative images of cells with EV-lipid accumulation at 3 or 24 h post-EV addition periods. Red fluorescence indicates EV-lipid. DNA was stained with Hoechst33342 (blue). Scale bars, 200 μm. **(E)** Accumulation levels of EV-lipid in 3D tumoroids. *P < 0.05, **P < 0.01, ***P < 0.001, *n* = 8 (biological replicates).

It was shown that ABCG1 plays a critical role in mediating cholesterol efflux to HDL and preventing cellular lipid accumulation (11). It was also shown that oxidized lipoproteins (57, 58) and phosphatidylcholine (59) were cytotoxic. These studies prompted us to examine whether depletion of ABCG1 could trigger the accumulation of EV-derived substances and alter the viability of tumor cells. We labeled the lipid membrane of LuM1-derived EVs with sphingolipid-associated red fluorescent material. We then investigated the accumulation of the EV-derived red fluorescence taken up by ABCG1-depleted or control LuM1 cells, firstly in the 2D-cultured system and then into 3D tumoroids. EV-lipids were accumulated to a greater extent in the ABCG1-depleted LuM1 cells compared with the control cells (Figures 5A,B). Such differences in the accumulation levels of EV-lipids between ABCG1-depleted and control cells were found in 3 h after the addition of EVs but were recovered in 24 h in the 2D cultured conditions (Figures 5C,D). We next investigated EV-lipid accumulation in 3D tumoroids with or without ABCG1-depletion. ABCG1 depletion significantly triggered the accumulation of EV-lipids in tumoroids until 24 h after EV addition compared with the control tumoroids (Figure 3E).

These findings suggested that depletion of ABCG1 triggered the accumulation of EV-derived lipids.

### Cancer prognostic value of ABCG1

**Figure 6:**
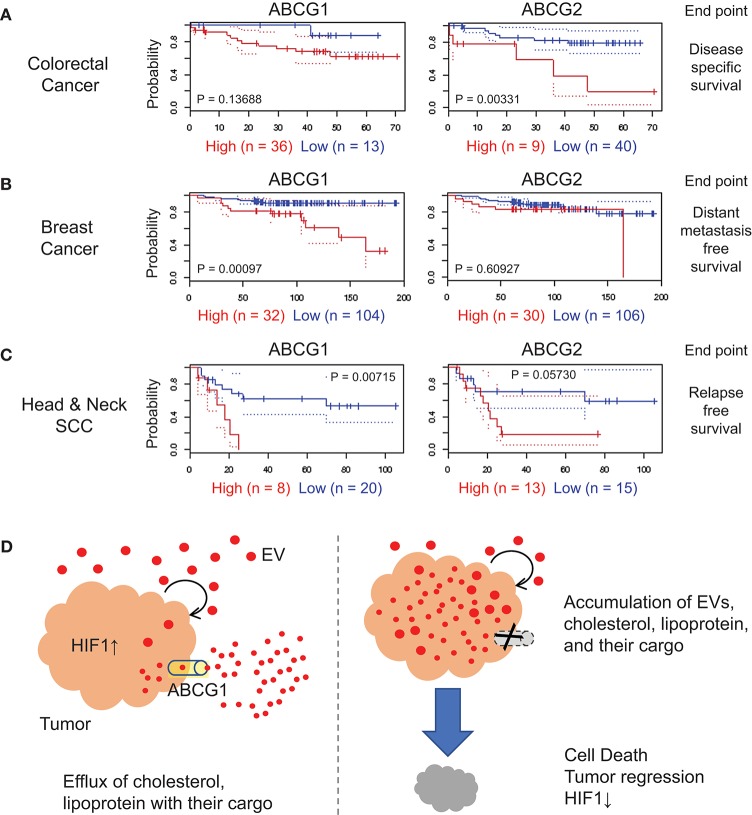
Correlation of ABCG1/G2 expression levels with cancer prognosis. ABCG1/G2 were analyzed by using PrognoScan and the Kaplan-Meier estimates of the indicated cohort studies were shown with minimum *P* values. The detailed data were shown in Table S1. Red, the high-level expression group. Blue, the low-level expression group. **(A)** colorectal cancer with an endpoint of disease-specific survival, **(B)** breast cancer with an endpoint of distant metastasis-free survival, and **(C)** head and neck squamous cells carcinoma with an endpoint of relapse-free survival. **(D)** Roles of ABCG1 in tumor progression and its targeting. Tumor cells secrete redundant metabolites with their EVs and lipoproteins, some of which may be toxic to themselves when accumulated in the tumor cells. ABCG1 plays an efflux role for lipoproteins, cholesterol, and their cargos that may include redundant and cytotoxic substances. The efflux and detoxification role of ABCG1 can promote cancer cell survival and tumor growth with elevated HIF1-α level. In our study, depletion of ABCG1 triggered the accumulation of EVs and death in tumor cells.

We next investigated the correlation of *ABCG1/2* gene expression in tumor clinical samples and prognosis of patients suffering from a range of cancer types, by searching public database PrognoScan (54), a tool for assessing the biological relationship between a large collection of publicly available cancer microarray datasets with clinical annotation. The correlation between *ABCG1/2* expression and poor prognosis of patients suffering from colorectal cancer was found with an endpoint of disease-specific survival (Figure 6A, Table S1), although this was not consistent among total nine cohort studies of colorectal cancer available in the meta-analysis. A significant correlation between *ABCG1* expression and poor prognosis in a breast cancer cohort study was found with an endpoint of distant metastasis-free survival with a minimum *P*-value 0.00097 (Figure 6B, Table S1). The correlation between *ABCG1* (*P* = 0.00715) or *ABCG2* (*P* = 0.0573) expression and poor prognosis of patients suffering from head and neck cancer was found with an endpoint of relapse-free survival (Figure 6C, Table S1). These data suggested that high expression of ABCG1 in tumor clinical samples could be a potential prognostic marker of metastatic breast cancer and recurrent head and neck squamous cell carcinomas.

### Genetic amplification of ABCG genes in resistant cancer

We also showed that ABCG1 and ABCG2 were co-expressed in the metastatic colon cancer cells. ABCG2 is also known as breast cancer resistant protein (BCRP) that plays an efflux role for anti-cancer drugs in chemoresistance (60, 61). To investigate genetic aberrations that could influence gene expression of these ABC genes, we searched alteration frequencies of ABC-G family among a range of cancer types in cBioPortal (62), a public portal database. Amplification of *ABCG* group genes was found in 55% of breast cancer patient-derived xenograft samples (16/29 samples) and in 28% of castration-resistant prostate cancer samples (Figure S7a). However, amplification of ABC-G genes were not found in colorectal cancer clinical samples. Among the five genes of the ABC-G group, *ABCG1* was the most frequently altered (Figures S7b–e). Therefore, genetic amplification and cancer cell aggregation with increased stemness can increase levels of ABC-G group proteins that can be correlated with and be useful for prognosis of cancer endocrine resistance.

## Discussion

We showed ABCG1 to be markedly expressed in metastatic colon cancer cells, their tumoroids as well as in subcutaneous tumors (Figures 1, 2). Genetic amplification of *ABCG1* is found in 10–35% of clinical samples of metastatic cancer cases (Figure S7). Coincident with increased secretion of EVs and accumulation of proteins, cellular ABCG1 was increased when tumoroids became enlarged (Figure 2). Depletion of ABCG1 reduced growth of the tumoroids (Figure 3) and lowered levels of HIF-1α around the central necrotic areas in tumors *in vivo* (Figure 4). This appears highly conceivable, inasmuch as the metastatic colon cancer cells, tumoroids and tumors derived from them express high level of ABCG1. Notably, depletion of ABCG1 increased the accumulation of EVs in the tumoroids and declined their viability inasmuch as potential loss of detoxification (Figures 3, 5, 6D). These findings suggest that ABCG1 plays a crucial role in tumor progression and that depletion of ABCG1 triggers the accumulation of EVs, lipoproteins, and their cargo and tumor regression.

Our study also touches upon an ABCG1-mediated mechanism underlying tumor progression. We showed that depletion of ABCG1 increased the accumulation of EVs, lipoproteins, and their potential cargos in the metastatic cancer tumoroids leading to reduction in their viability (Figures 3, 5). ABCG1 and ABCA1 plays a critical role in mediating efflux of cholesterol and phospholipids, removing excess cholesterol and phospholipids from cells, and preventing cellular lipid accumulation (10, 11). Cholesterol and phospholipids are essential to the body and cells, but an excess of cholesterol or lipids is toxic and a risk factor for arteriosclerosis (63), and is presumably toxic for tumors as well. Reactive oxygen species generate oxidized low-density lipoproteins (oxLDL) (64) and oxidized lipoproteins and phosphatidylcholine are cytotoxic (57–59). Thus, we conjectured that that tumors are protected from toxic lipid products by ABCG1-mediated detoxification (Figure 6D).

The 3D hypoxic tumoroids grow very slowly but produced robust levels of ABCG1 and EVs in stemness-enhancing milieu, indicating a reprogrammed metabolism in tumoroids, also suggested in our previous studies (2). Depletion of ABCG1 led to reduced HIF-1α levels around the central necrotic area in tumors. It was shown that HIF-1α is increased in the central areas in tumors, where this transcription factor activates glycolysis gatekeeper PDK1 gene leading to enhancing glycolysis and subsequent induction of reprogramming factors (38). Surprisingly, ABCG1 is an upstream regulator of such a crucial signaling of HIF-1α. We first oppositely hypothesized that HIF-1α could activate ABCG1 gene transcription inasmuch as two HIF-1α-binding sites were found in ABCG1 gene promoter region; however, this retrograde pathway arose from our present studies. ABCG1 was expressed not only hypoxic area but broad area in tumoroids and tumors and our results suggest the oppositely aligned regulatory axis ABCG1-to-HIF1. It was shown that extracellular HDL binding to scavenger receptor SR-B1 promotes nuclear translocation of HIF-1α via the PI3K-Akt pathway (65), which may be activated in tumors. We are currently investigating further mechanisms underlying ABCG1-mediated HIF1 activation and tumor growth.

Analyzing PrognoScan database indicated that elevated expression of *ABCG1* and *ABCG2* were correlated with poor prognosis of patients suffering from colorectal cancer and head and neck squamous cell carcinoma. We also found that *Abcg2 / Bcrp* mRNA level was increased in the rapidly metastatic, aggregative LuM1 cells as compared to the slowly metastatic parental Colon26 cells, although the absolute expression level of *Abcg2* was very low (Figures 1A,C). It has been shown that HIF-1α trans-activates *ABCG2* gene under hypoxia (66, 67). Therefore, it is conceivable that *Abcg2* expression was induced in the hypoxic aggregation of cancer cells. It was also shown that ABCG2 level was increased in ovarian tumor-initiating cells (TIC/CIC/CSC) and depletion of either HIF-1α or ABCG2 reduced tumorsphere (cell aggregate) formation, demonstrating HIF-1α-to-ABCG2 axis in hypoxic tumorsphere (68). We showed that depletion of ABCG1 reduced HIF-1α around the necrotic area of a tumor. Therefore, it is conceivable that ABCG1 depletion could also reduce *Abcg2* expression level through reduction of HIF-1α (the hypothetical regulatory axis is ABCG1>HIF-1α>ABCG2). Depletion of ABCG1 reduced ABCG2 mRNA level, but this was not siRNA-concentration-dependent manner, suggesting a complex regulatory mechanism (Figure S6). It has been shown that ABCG2 is a drug efflux pump that involves chemoresistance in cancer (60, 61, 69, 70). Depletion of the drug efflux pump ABCG2 increased chemo-sensitivity to paclitaxel, cisplatin, and imatinib through intracellular accumulation of these drugs (68). Distinctively, ABCG1 is a lipid efflux pump. We demonstrated that depletion of ABCG1 triggered the intracellular accumulation of EV-lipid and reduction of the cancer cell viability. Intratumoral accumulation of EV-lipid may be cytotoxic.

We approached tumor milieu using stemness-enhancing medium with 3D culture milieu, which increased tumorigenesis as well as intra-tumoral hypoxia *in vitro*. In addition, acidic milieu are particular traits that increase tumor malignancy (71–73). Acidic milieu is caused by increased extracellular proton and lactate. A macrophage lineage osteoclasts also create an acidic extracellular milieu by secretion of protons via vacuolar H^+^-ATPase (v-ATPase) proton pumps during bone resorption. Bone-colonized cancer cells also release protons and lactate via plasma membrane pH regulators to avoid intracellular acidification resulting from increased aerobic glycolysis known as the Warburg effect (74). Activities of proton pumps increase acidity in tumor milieu and in intracellular vesicles such as lysosomes and phagosomes, from which multi-vesicular bodies (MVB) and exosomes are generated. Therefore, the acidic milieu could increase exosome biosynthesis and secretion via altering the properties of lysosomes and MVBs. Indeed, microenvironmental acidosis was shown to stimulate cancer cells to secrete exosomes, an effect which were inhibited by proton pump inhibitors (PPIs) (75, 76). In our studies, cancer cell aggregation also triggered secretion of EVs (Figure 2) (2). Hence, we are currently challenging control and relation of aerobic glycolysis that generates lactate acidic milieu, acidity in intracellular vesicles, and molecular and secretory traits of EVs. Glycolysis is likely to be amplified in the hypoxic cores of the tumoroids, while diffusion of lactate out of these structures may be slower due to the increased cell density.

ABCG1-mediated cholesterol efflux may involve lysosomal activity / acidity that promote exosome secretion. ABCG1 and ABCA1 pump cholesterol out of cells (77) and therefore depletion of the efflux pump ABCG1/A1 can result in lysosomal accumulation of free cholesterol (FC). Increasing levels of lysosomal membrane FC were shown to inhibit lysosomal acidification (78). It was demonstrated that excess FC in the lysosomal membrane leads to loss of acidity as a result of inhibition of v-ATPase proton pumping activity in the lysosomal membrane. Therefore, depletion of ABCG1 may trigger intra-vesicular accumulation of FC, which inhibit v-ATPase activity and reduce lysosomal acidity. Interestingly, PPIs also have a pro-apoptotic effect to cancer cells (79), implicating a potential accumulation mechanism similar with ABCG1-depletion.

EVs or nanovesicles such as exosomes and more canonically liposomes have been broadly proposed for application and ability to deliver anti-cancer and anti-disease drugs, including theranostic molecules (80–85). However, we recently showed that anti-EGFR therapeutic antibody cetuximab is secreted with EVs by oral cancer cells (4), suggesting that cancer cells can secrete toxic and redundant substances within EVs as a novel mechanism underlying drug resistance. Low pH within melanomas was also shown to promote the release of cisplatin-contained exosomes, which PPI inhibited, suggesting a potent role for acidic milieu in chemoresistance (86, 87). Not only cancer cells but also tumor-associated macrophages can express ABCG1 that pump cholesterol out, implicating that ABCG1 in tumor milieu may also play a detoxifying role. However, delivery of ABCG1-targeting siRNA with EVs/nanovesicles may overcome such EV-mediated drug resistance. Our allograft experiments and 3D tumoroid model have contributed to deeper understanding and may be an important model for the tumor milieu. We are currently developing a strategic method to deliver ABCG-targeting siRNA with nanovesicles / EVs to tumors and their acidic milieu.

In conclusion, we showed that (i) ABCG1 was robustly expressed in the metastatic aggregative cells, in tumors derived from them, and in some types of clinical cancer correlated with poor prognosis of patients; (ii) depletion of the ABCG1 pump triggered the autocrine accumulation of EVs and cancer cell death in tumoroids, indicating a novel therapeutic strategy by which redundant and toxic substances may be accumulated in tumors by ABCG1-depletion, leading to their regression.

## Author contributions

TE and KK conceptualized and designed the study. YN, YO, and TE performed bioinformatics. KK, TE, MT, JM, CS, HK, KuO and JA prepared resources. CS, EA, and TE devised methodology. CS, YN, YO, HK, MI, KiO, and KaO carried out the experimentation. YN, CS and HK performed formal analysis. TE, CS, YN, and HK interpreted data. TE wrote the manuscript. YN, SC, JM, KuO, and TE revised and edited the manuscript. All authors reviewed the manuscript.

### Conflict of interest statement

The authors declare that the research was conducted in the absence of any commercial or financial relationships that could be construed as a potential conflict of interest. The reviewer AR and the handling editor declared their shared affiliation.

## Supplementary material

Figure S1 Tumorigenicity, a rapidly lung-metastatic ability, and an aggregative property of the LuM1 cells. LuM1 and Colon26 cells (500 k cells) were subcutaneously injected into side abdominal walls of BALB/c mice. Primary tumors **(a)** and lungs **(b)** were resected on day 35 post-injection period. Arrowheads indicate typical metastatic nodules. Scale bar, 5 mm. **(c)** Tumor incidence and lung metastasis of Colon26 and LuM1 cells. Data were expressed as mean ± SEM.

Figure S2 Morphological and size analyses of EVs. **(a)** Representative TEM images of EVs secreted by LuM1 cells. Scale bars, 50 μm. **(b)** Particle diameter distribution of LuM1-EVs.

Figure S3 RT-qPCR analysis to find an appropriate internal control and of *Abcg1* knockdown. **(a)** Relative mRNA levels of internal control candidates. The vertical axes indicate relative expression levels. Higher values mean the absolute expression levels were higher. LuM1 cells were cultured in no reagent control medium or control siRNA transfected condition with the indicated concentrations. Reference cDNA standards were prepared from the cDNA pool of the LuM1 cells by step dilution. **(b)** RT-qPCR analysis of *Abcg1*. The *Abcg1* expression levels relative to Hprt1 or Hplp0 were shown. *n* = 3, ^*^*P* < 0.05, ^**^*P* < 0.001.

Figure S4 Optimization of electroporation-transfection to LuM1 cells. LuM1 cells (550k cells) were transfected with 10 μg of pCMV-GFP with the indicated 10 different electroporation conditions. **(a)** Representative fluorescence images with GFP at 24 h post-electroporation period. **(b)** A list of electroporation conditions, gene transfer efficiencies, and cell viabilities.

Figure S5 Full images of Western blot analysis of ABCG1 and GAPDH at day 5 post-electroporation transfection.

Figure S6 Altered aggregation of LuM1 with or without ABCG1 depletion. LuM1 cells were transfected with control or ABCG1-targeting siRNA by using electroporation method and seeded at the concentration of 4,000 cells per well in a 96-well 3D cell culture plate (NCP). **(a)** Box-and-whisker plot analysis of hypoxic cell aggregates. A hypoxia probe Lox-1 was added on day 7 post-transfection period and the cell aggregates were scanned on day 8. **(b)** Stacked bar graph showing rate of cell aggregates sized >5,000 μm^2^ (black), 1,000–5,000 μm^2^ (gray), and 300–1,000 μm^2^ (white). Control si group, *n* = 143, Abcg1 si group, *n* = 111. **(c,d)** ABCG1 and ABCG2 mRNA levels upon transfection of 50 nM or 100 nM siRNA-ABCG1 or -control. The mRNA levels were normalized with *Gapdh*** (c)** or *Hprt1*** (d)**. ^*^*P* < 0.05, ^**^*P* < 0.01. n.s., not significant. **(c)** *n* = 4 (biological quadruplicate), **(d) ***n* = 3 (biological triplicate).

Figure S7 Genetic alteration of ABC-G group in cancer. The data were obtained by searching cBioPortal. **(a–c)** A combined study was carried out containing samples from 169 studies. **(a)** Total alteration frequency of ABCG group (ABCG1, G2, G4, G5, and G8) among the 169 cancer studies. **(b,c)** Alteration frequencies of ABCG1 **(b)** and ABCG2 **(c)** among the 169 cancer studies. **(d)** Genetic alteration of each ABCG gene in samples of breast cancer xenograft and metastasis. Alteration frequencies in a combination of these two studies (left) and of the PDX study only (right) were indicated. **(e)** Genetic alteration of each ABCG gene in castration-resistant neuroendocrine prostate cancer samples.

Table S1 Correlation between ABCG1/G2 expression and prognosis of patients suffering from colorectal cancer, breast cancer, and head and neck cancer were investigated using the PrognoScan.

## Abbreviations

ABC: ATP-binding cassette
CIC: cancer initiating cell
CSC: cancer stem-like cell
EV: extracellular vesicle
HIF: hypoxia-inducible factor
TIC: tumor initiating cell.
