# Supplementary material for: Depletion of Lipid Efflux Pump ABCG1 Triggers the Intracellular Accumulation of Extracellular Vesicles and Reduces Aggregation and Tumorigenesis of Metastatic Cancer Cells
Source: Front Oncol. 2018 Oct 10;8:376. doi: 10.3389/fonc.2018.00376 (PMC6191470; doi:10.3389/fonc.2018.00376)
Supplement: Figure S1 — Tumorigenicity, a rapidly lung-metastatic ability, and an aggregative property of the LuM1 cells. LuM1 and Colon26 cells (500 k cells) were subcutaneously injected into side abdominal walls of BALB/c mice. Primary tumors (a) and lungs (b) were resected on day 35 post-injection period. Arrowheads indicate typical metastatic nodules. Scale bar, 5 mm. (c) Tumor incidence and lung metastasis of Colon26 and LuM1 cells. Data were expressed as mean ± SEM. [file Data_Sheet_1.pdf]

Figure S1

a

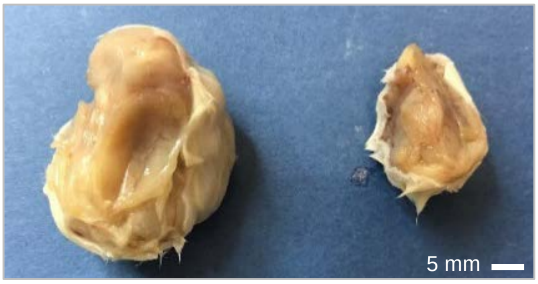

b

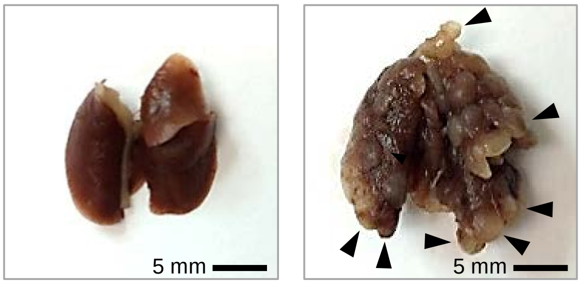

c

	Colon26	LuM1
Tumor incidence	1/5 (20%)	3/4 (75%)
Number of metastatic nodules	1.0 ± 1.0 (n = 5)	17.8 ± 9.9 (n=4)
Number of metastatic nodules in cases of metastases	5.0 (n = 1)	23.7 ± 11.3 (n = 3)

Figure S2

a

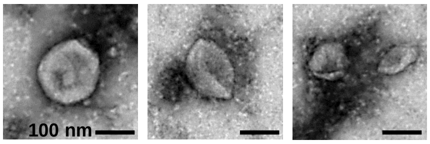

100 nm

b

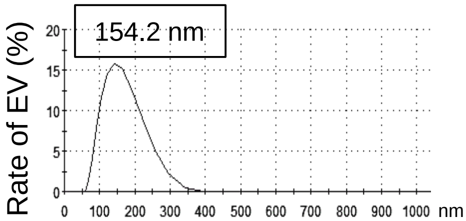

154.2 nm
Rate of EV (%)
20
15
10
5
0
0
100
200
300
400
500
600
700
800
900
1000
nm

Figure S3

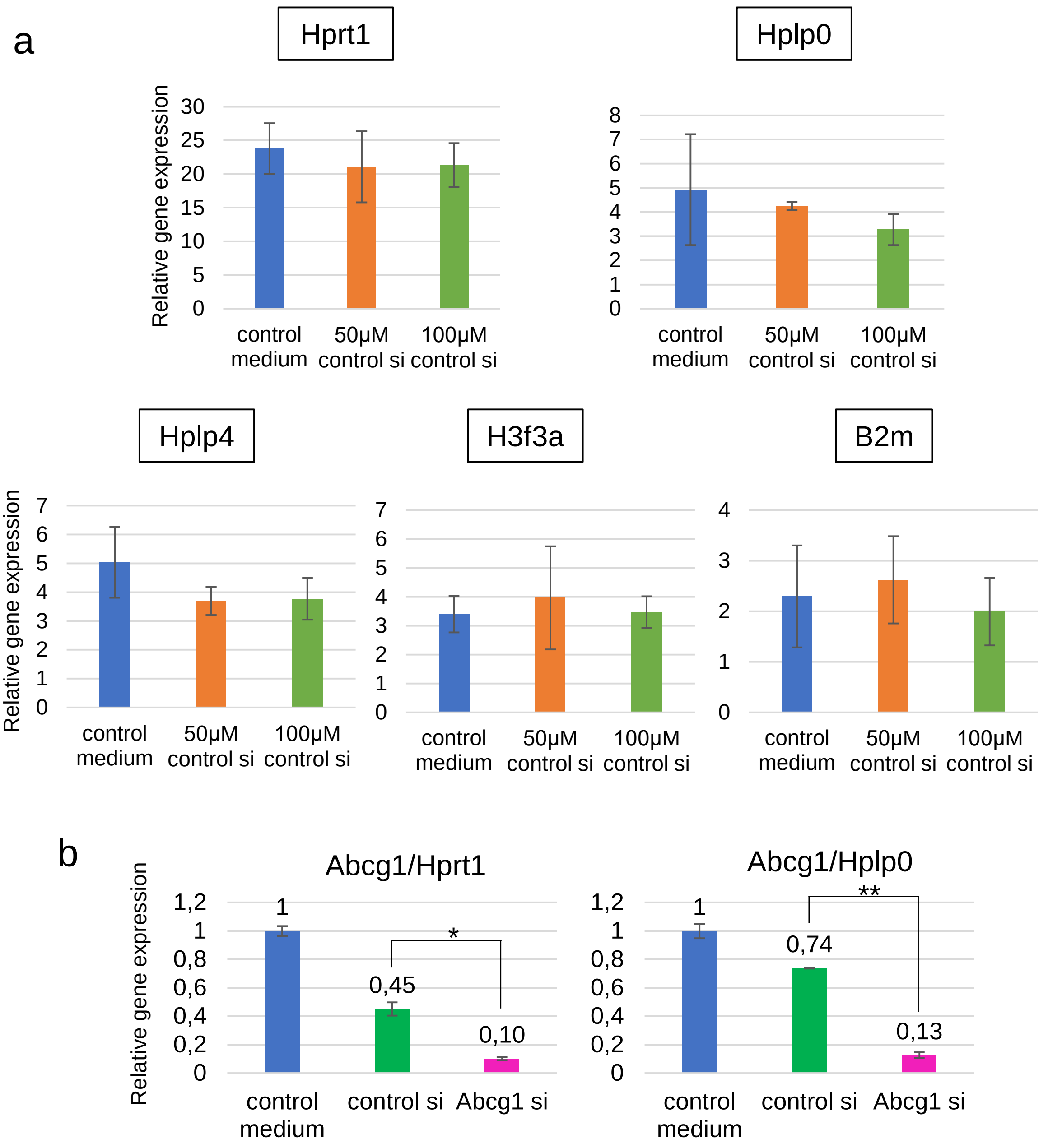
a
Hprt1
Relative gene expression
30
25
20
15
10
5
0
control medium
50µM control si
100µM control si
Hplp0
8
7
6
5
4
3
2
1
0
control medium
50µM control si
100µM control si
Hplp4
Relative gene expression
7
6
5
4
3
2
1
0
control medium
50µM control si
100µM control si
H3f3a
7
6
5
4
3
2
1
0
control medium
50µM control si
100µM control si
B2m
4
3
2
1
0
control medium
50µM control si
100µM control si
b
Abcg1/Hprt1
Relative gene expression
1,2
1
0,8
0,6
0,4
0,2
0
1
0,45
0,10
*
control medium
control si
Abcg1 si
Abcg1/Hplp0
1,2
1
0,8
0,6
0,4
0,2
0
1
0,74
0,13
**
control medium
control si
Abcg1 si

Figure S4

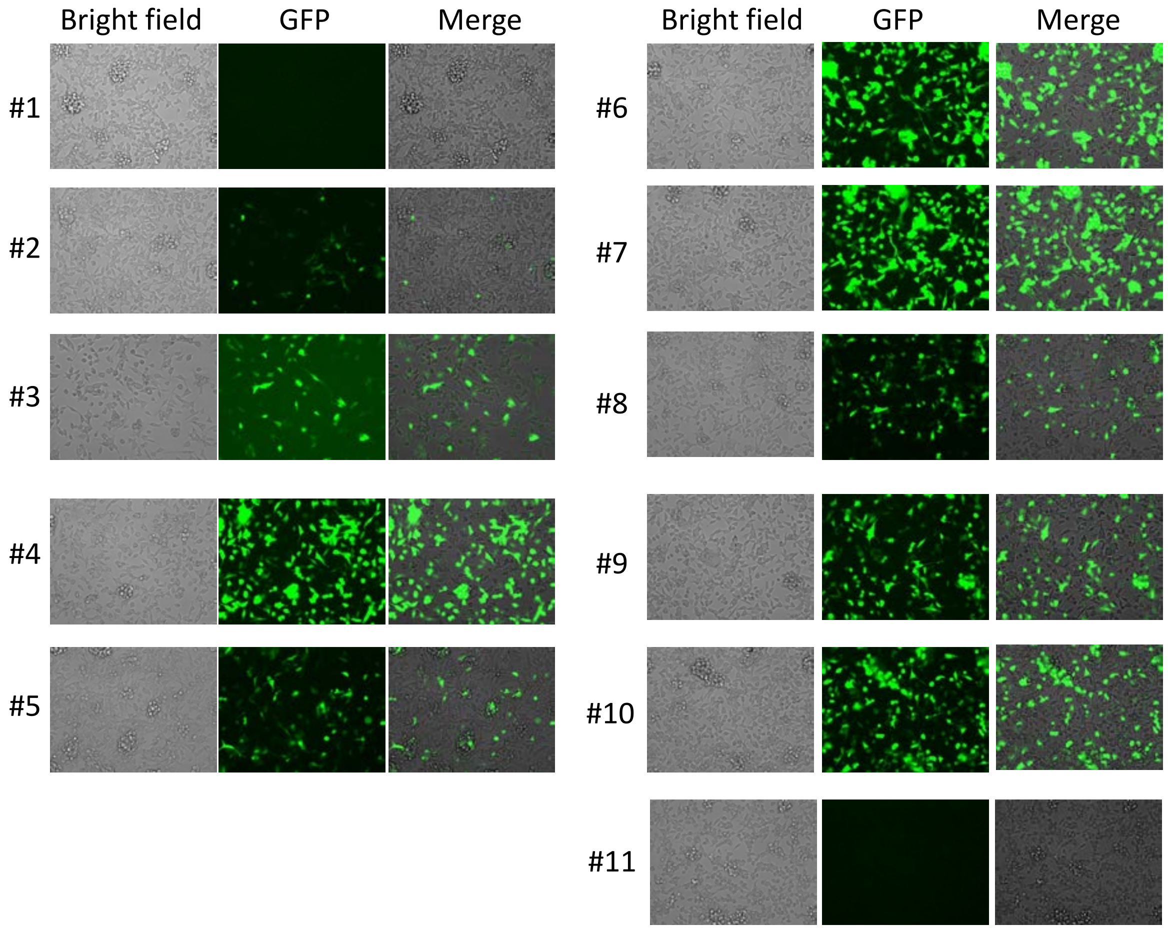

Bright field
GFP
Merge
#1
#2
#3
#4
#5
Bright field
GFP
Merge
#6
#7
#8
#9
#10
#11

Figure S5

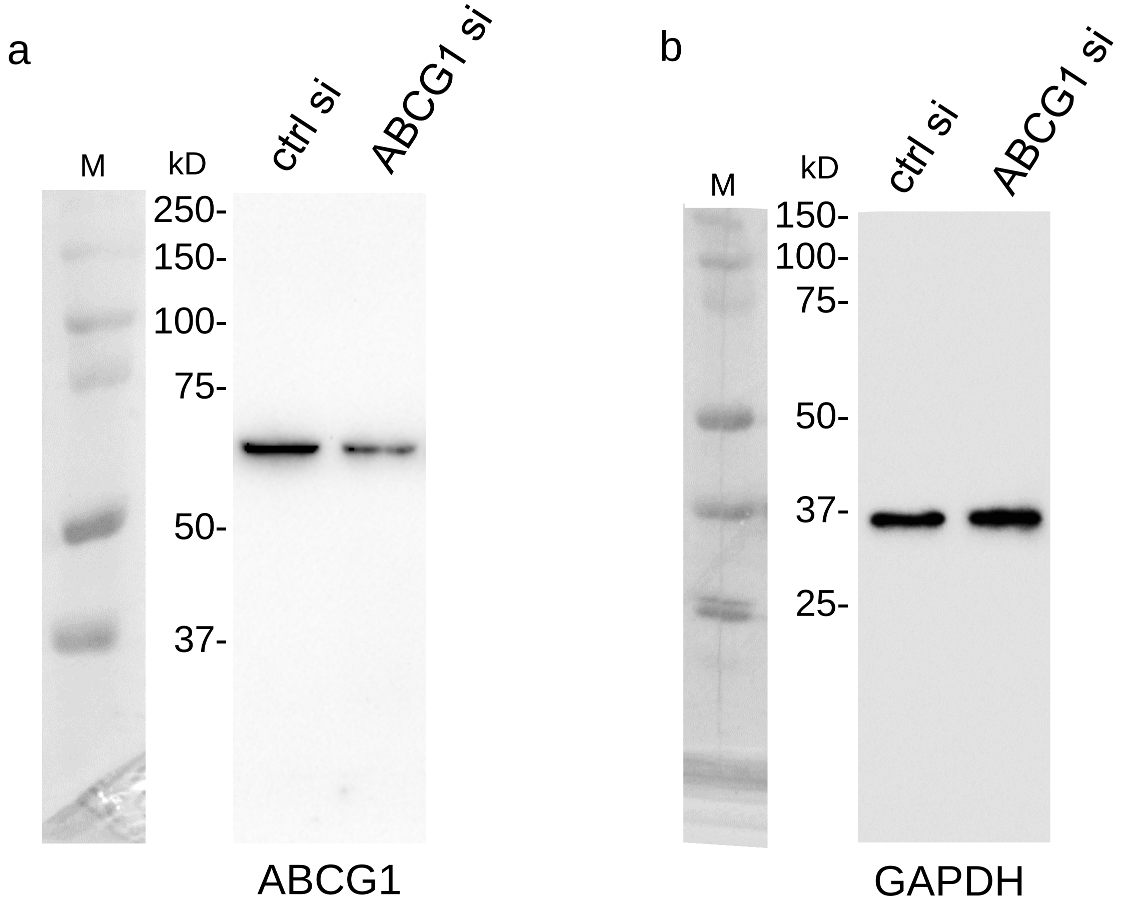
a
M
kD
ctrl si
ABCG1 si
250-
150-
100-
75-
50-
37-
ABCG1
b
M
kD
ctrl si
ABCG1 si
150-
100-
75-
50-
37-
25-
GAPDH

Figure S6

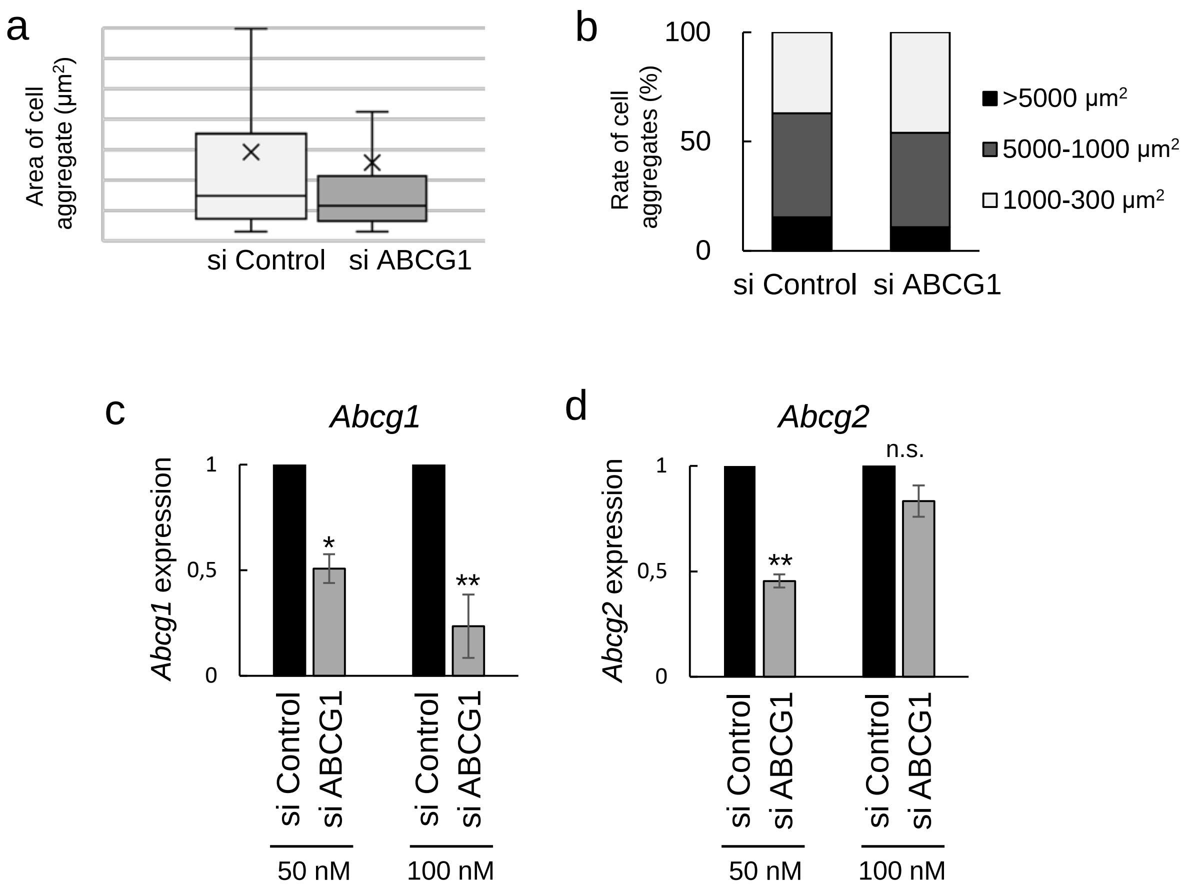
a
Area of cell aggregate (μm2)
si Control
si ABCG1
b
Rate of cell aggregates (%)
100
50
0
>5000 μm2
5000-1000 μm2
1000-300 μm2
si Control
si ABCG1
c
Abcg1
Abcg1 expression
1
0,5
0
*
**
si Control
si ABCG1
si Control
si ABCG1
50 nM
100 nM
d
Abcg2
Abcg2 expression
n.s.
1
0,5
0
**
si Control
si ABCG1
si Control
si ABCG1
50 nM
100 nM

Figure S7

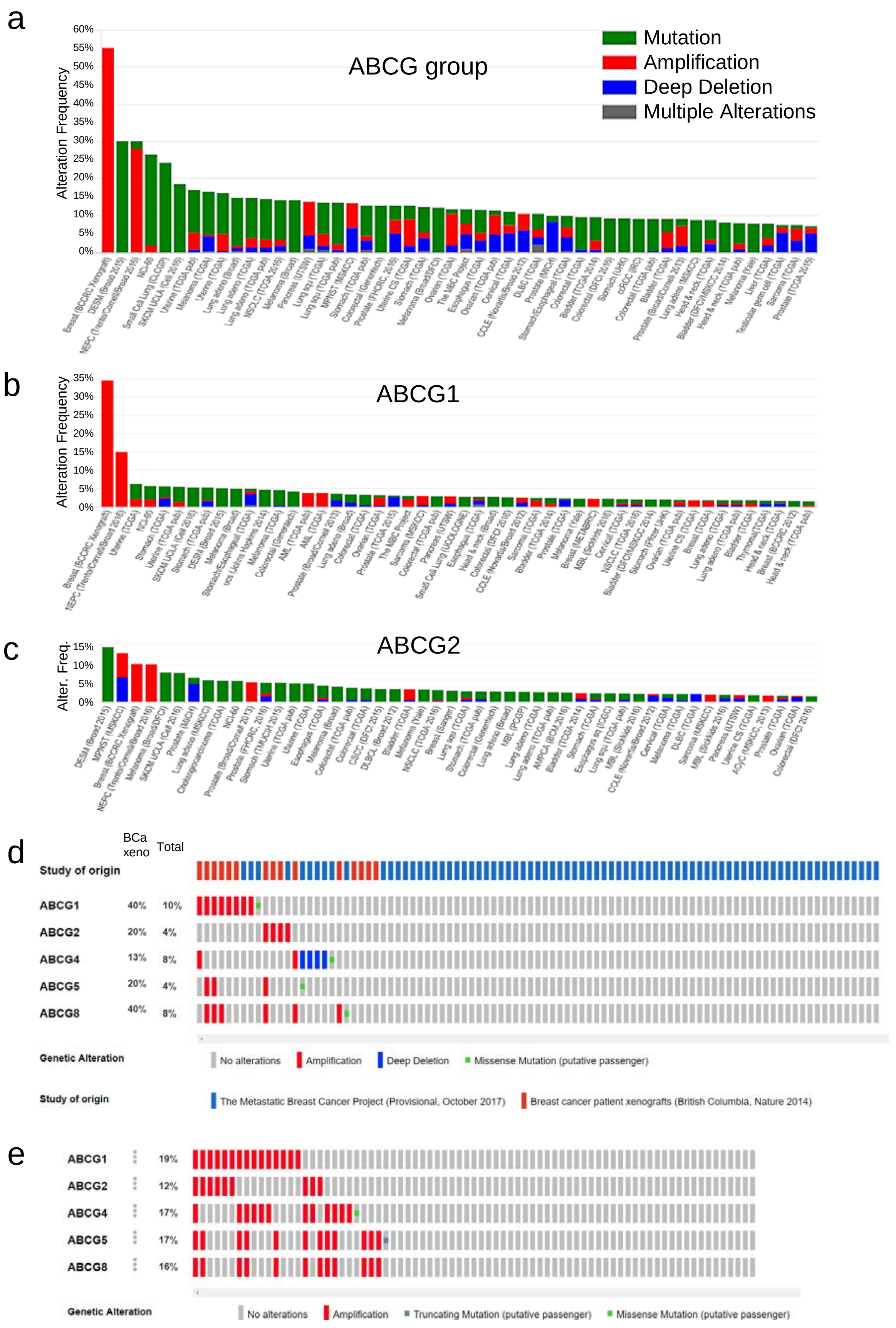

a
ABCG group
Mutation
Amplification
Deep Deletion
Multiple Alterations
Alteration Frequency
60%
55%
50%
45%
40%
35%
30%
25%
20%
15%
10%
5%
0%
b
ABCG1
Alteration Frequency
35%
30%
25%
20%
15%
10%
5%
0%
c
ABCG2
Alter. Freq.
15%
10%
5%
0%
d
BCa xeno
Total
Study of origin
ABCG1
40%
10%
ABCG2
20%
4%
ABCG4
13%
8%
ABCG5
20%
4%
ABCG8
40%
8%
Genetic Alteration
No alterations
Amplification
Deep Deletion
Missense Mutation (putative passenger)
Study of origin
The Metastatic Breast Cancer Project (Provisional, October 2017)
Breast cancer patient xenografts (British Columbia, Nature 2014)
e
ABCG1
19%
ABCG2
12%
ABCG4
17%
ABCG5
17%
ABCG8
16%
Genetic Alteration
No alterations
Amplification
Truncating Mutation (putative passenger)
Missense Mutation (putative passenger)
