# Supplementary material for: Depletion of Lipid Efflux Pump ABCG1 Triggers the Intracellular Accumulation of Extracellular Vesicles and Reduces Aggregation and Tumorigenesis of Metastatic Cancer Cells
Source: Front Oncol. 2018 Oct 10;8:376. doi: 10.3389/fonc.2018.00376 (PMC6191470; doi:10.3389/fonc.2018.00376)
Supplement: Table S1 — Correlation between ABCG1/G2 expression and prognosis of patients suffering from colorectal cancer, breast cancer, and head and neck cancer were investigated using the PrognoScan. [file Table_1.docx]

**Table S1.** Correlation between ABCG1/G2 expression and prognosis of patients suffering from colorectal cancer, breast cancer and head and neck cancer were investigated using the PrognoScan.

| CANCER TYPE (SUBTYPE) | Colorectal cancer | Colorectal cancer | Breast cancer | Breast cancer | Head and neck cancer (SCC) | Head and neck cancer (SCC) |
| --- | --- | --- | --- | --- | --- | --- |
| ID_NAME | ABCG1 | ABCG2 | ABCG1 | ABCG2 | ABCG1 | ABCG2 |
| DATASET | GSE17537 | GSE17537 | GSE12093 | GSE12093 | GSE2837 | GSE2837 |
| N | 49 | 49 | 136 | 136 | 28 | 28 |
| ENDPOINT | Disease Specific Survival | Disease Specific Survival | Distant Metastasis Free Survival | Distant Metastasis Free Survival | Relapse Free Survival | Relapse Free Survival |
| COHORT | VMC | VMC | IO, NCI, TUM, CCF (1992-2000) | IO, NCI, TUM, CCF (1992-2000) | VUMC, VAMC, UTMDACC (1992-2005) | VUMC, VAMC, UTMDACC (1992-2005) |
| ARRAY TYPE | HG-U133_Plus_2 | HG-U133_Plus_2 | HG-U133A | HG-U133A | U133_X3P | U133_X3P |
| PROBE ID | 204567_s_at | 209735_at | 204567_s_at | 209735_at | g8051574_3p_a_at | g12414050_3p_at |
| CONTRIBUTOR | Smith | Smith | Zhang | Zhang | Chung | Chung |
| CUTPOINT | 0.265306 | 0.816327 | 0.764706 | 0.779412 | 0.714286 | 0.535714 |
| MINIMUM P-VALUE | 0.136888 | 0.00331325 | 0.00097169 | 0.207877 | 0.00715101 | 0.0573061 |
| CORRECTED P-VALUE | - | 0.0682319 | 0.0248784 | - | 0.12506 | - |
| ln(HR-high / HR-low) | 1.42958 | 1.57101 | 1.37998 | 0.609279 | 1.4748 | 1.04415 |
| COX P-VALUE | 0.714539 | 0.63975 | 0.00487242 | 0.793331 | 0.134986 | 0.755988 |
| ln(HR) | 0.140739 | 0.152763 | 1.39334 | 0.131701 | 0.461254 | -0.158272 |
| HR [95% CI-low CI-upp] | 1.15 [0.54 - 2.45] | 1.17 [0.61 - 2.21] | 4.03 [1.53 - 10.63] | 1.14 [0.43 - 3.06] | 1.59 [0.87 - 2.90] | 0.85 [0.31 - 2.32] |
